# Evaluation of P2X7 Receptor Function in Tumor Contexts Using rAAV Vector and Nanobodies (AAVnano)

**DOI:** 10.3389/fonc.2020.01699

**Published:** 2020-09-11

**Authors:** Mélanie Demeules, Allan Scarpitta, Catalina Abad, Henri Gondé, Romain Hardet, Carolina Pinto-Espinoza, Anna Marei Eichhoff, Waldemar Schäfer, Friedrich Haag, Friedrich Koch-Nolte, Sahil Adriouch

**Affiliations:** ^1^Normandie University, UNIROUEN, INSERM, U1234, Pathophysiology, Autoimmunity, Neuromuscular Diseases and Regenerative THERapies, Rouen, France; ^2^Institute of Immunology, University Medical Center Hamburg-Eppendorf, Hamburg, Germany

**Keywords:** P2X7, tumor, Adeno-associated virus, Nanobodies (VHH), animal models

## Abstract

Adenosine triphosphate (ATP) represents a danger signal that accumulates in injured tissues, in inflammatory sites, and in the tumor microenvironment. Extracellular ATP is known to signal through plasma membrane receptors of the P2Y and P2X families. Among the P2X receptors, P2X7 has attracted increasing interest in the field of inflammation as well as in cancer. P2X7 is expressed by immune cells and by most malignant tumor cells where it plays a crucial yet complex role that remains to be clarified. P2X7 activity has been associated with production and release of pro-inflammatory cytokines, modulation of the activity and survival of immune cells, and the stimulation of proliferation and migratory properties of tumor cells. Hence, P2X7 plays an intricate role in the tumor microenvironment combining beneficial and detrimental effects that need to be further investigated. For this, we developed a novel methodology termed AAVnano based on the use of Adeno-associated viral vectors (AAV) encoding nanobodies targeting P2X7. We discuss here the advantages of this tool to study the different functions of P2X7 in cancer and other pathophysiological contexts.

## Introduction

Adenosine triphosphate (ATP) released into the extracellular space represents a well-known danger signal that can signal through two main families of plasma membrane receptors: G protein-coupled receptors, named P2Y receptors, and ATP-gated ion channels termed P2X receptors (1). Among the latter family, P2X7 (also known as P2RX7) forms a homotrimeric receptor that has attracted much interest in the fields of inflammation and cancer. Activation of P2X7 by relatively high concentrations of extracellular ATP leads to Na^+^ and Ca^2+^ influx, and K^+^ efflux. This triggers not only major changes in the cellular ionic content, but also signaling and metabolic pathways involved in cell activation, survival and fate. Prolonged activation of P2X7 leads to the opening of a membrane pore allowing the entry of large molecules of up to 900 Da (2). Whether this membrane permeabilization is due to dilation of the P2X7 channel itself, or the activation of non-selective pores like pannexin-1, gasdermin-D, or anoctamin-6, may depend on the cellular context, the lipid composition of the membrane, and on the level of expression of these proteins (2–4). Whatever the precise molecular mechanism leading to pore formation, P2X7 activation can lead to major perturbation of intracellular ion balance and to modification of cellular activities, cellular functions, and cell fate.

High concentrations of extracellular ATP, released in the vicinity of stressed or damaged cells, during inflammation, but also within the tumor site, represent a prototypic “danger signal” that can influence the activity and function of immune cells (5–8). P2X7 is expressed at the cell surface of various leukocytes, in particular monocytes, macrophages, T cells and notably regulatory T cells (Tregs), and is found also at the surface of numerous tumor cell types. Extracellular ATP plays a complex role within the tumor microenvironment depending on multiple factors such as its concentration, the abundance of ecto-ATPases, the expression level of P2X7, and the nature of the P2X7 variant expressed by immune and tumor cells (9).

The functions of P2X7 in immune cells are largely documented. Gating P2X7 on macrophage, microglia, and dendritic cells triggers the formation of the inflammasome, a multiprotein complex that drives caspase-mediated maturation and release of the proinflammatory cytokines IL-1β and IL-18 (10). On T lymphocytes, P2X7 induces the shedding of CD62L and CD27 by metalloproteases (11, 12) and controls the differentiation, proliferation and survival of tissue resident memory T cells (13, 14). Regulatory T cells are known to express P2X7 at high levels, and its activation induces shedding of CD62L and CD27, phosphatidylserine exposure, pore formation, and finally leads to cell death (11, 15). Taken together, activation of P2X7 on myeloid and lymphoid cells converge to promote and amplify inflammation (16, 17). Animals deficient for P2X7 show reduced inflammation in several animal models including experimental colitis (18), collagen-induced arthritis (19), and experimental autoimmune encephalomyelitis (EAE) (20). P2X7 receptor antagonists have been developed by several pharmaceutical companies as potential novel anti-inflammatory drugs (21).

## Role of P2X7 on Tumor Growth and on Anti-Tumor Immune Responses

P2X7 functions in cancer are complex and depend on the composition of the tumor microenvironment and the nature of the cells that express this ion channel. P2X7 is associated with a pro-inflammatory activity which could be beneficial for tumor growth and maintenance. However, P2X7 activation is also linked to immunogenic cell death and has been associated with inflammasome formation and dendritic cell activation, which in turn promote adaptive anti-tumor immune responses (22). As mentioned earlier, P2X7 promotes Treg depletion, which increases antitumor immune responses by unleashing effector functions of T cells (6, 15). In line with the anti-tumor activity of P2X7, tumor cell lines appear to grow faster when transferred to P2X7^–/–^ mice (23), and genetic and pharmacological inactivation of P2X7 increases tumorigenesis in the colitis-associated cancer model (18). However, P2X7 is also expressed by most tumor cells, where it is associated with cancer progression. Indeed, tumor cells express P2X7 at low levels and/or express P2X7 variants that are inefficient to promote cell death but which instead exert a trophic activity. In such situations, tonic stimulation of P2X7 by extracellular ATP already present in the tumor microenvironment can stimulate cell metabolism, influences the Ca^2+^ content of mitochondria and endoplasmic reticulum, increases the intracellular ATP content, protects against apoptosis, and promote tumor growth (24–27). Accordingly, stable transfection of the colon carcinoma tumor model CT26 with a P2X7 expression vector accelerated tumor growth *in vivo* (28), and P2X7 blockade using oxidized ATP (oATP), a poorly selective but irreversible antagonist, reduced melanoma B16 tumor growth *in vivo* (29). Taken together, on the side of the tumor, P2X7 promotes cell survival, tumorigenic potential and proliferation, but on the side of immune cells, P2X7 favors dendritic cell activation, presentation of tumor antigens, and initiation of an adaptive immune response. It appears therefore that the net effect of P2X7 is difficult to predict and that the balance could be tilted toward a pro- or anti-tumorigenic outcome, depending on the composition of the tumor microenvironment, on the level of P2X7 expression, and on the nature and functionality of the P2X7 variants expressed by tumor cells.

## Methods to Study the Role of P2X7 *in vivo*

To investigate the role of P2X7 *in vivo*, several tools have been developed including genetically deficient animals, pharmacological inhibitors, and specific antibodies. P2X7^–/–^ mice have been widely used to study P2X7 function *in vivo* since the generation of the first two P2X7 KO strains by Pfizer and Glaxo (30, 31). However, it subsequently became apparent that both lines are leaky: the Glaxo line, in which exon 1 was targeted, still expresses the P2X7k variant on T cells (32, 33) while the Pfizer line, in which exon 13 was targeted, still expresses a C-terminal truncated variant displaying lower functionality (34). This situation could explain reported phenotypic and functional differences as well as conflicting results in disease models using these two P2X7 deficient lines (20, 35, 36). However, novel knockout models and P2X7-floxed mutants derived from the European Mutant Mouse Archive (EMMA) are now available and may facilitate the reevaluation of P2X7 functions *in vivo* in different disease models (37, 38).

Pharmacological inhibitors of P2X7 like brilliant blue G (BBG) and oATP have shown therapeutic benefit in several animal models including EAE, experimental colitis, inflammatory pain in arthritic animals, or/and antibody-mediated nephritis (36, 39–42). Blocking P2X7 by these small molecule inhibitors has also been shown to inhibit tumor growth in several tumor models that express P2X7 (28, 29). BBG and oATP are rather inexpensive but lack specificity and are associated with off-target side effects. The development of more specific antagonists by several pharmaceutical companies has facilitated the evaluation and confirmation of the role of P2X7 in these diseases (23, 28, 36, 39). However, these antagonists are sometimes more difficult to obtain and are expensive to use, notably in chronic models where they have to be injected every other days for several weeks.

## P2X7 Modulating Nanobodies

Antibodies represent another emerging class of potent pharmaceutical modulators that are used to block or to potentiate their targets *in vivo*. Conventional antibodies are composed of two heavy and two light chains that contribute together to the antigen-binding site. Conventional antibodies represent relatively large molecules of ∼150 kDa. In direct relation with their size, their biodistribution into tissues is limited (43). In comparison, smaller proteins derived from antibodies but containing only the binding domains fused directly via a linker (i.e., single-chain variable fragment or scFv) offer better biodistribution coefficients. More recently developed nanobodies offer an interesting alternative. Nanobodies are derived from unconventional antibodies found in llamas and other camelids that are composed only of heavy chains. Nanobodies and heavy chain antibodies present similar specificities and affinities as classical antibodies but exhibit a smaller size (80 kDa, 15 kDa) and a better tissue penetration notably into tumors (44–46).

A set of nanobodies modulating P2X7 was selected by phage display from llamas immunized with cDNA expression vectors or P2X7-transfected HEK cells encoding for mouse and human P2X7 (47). In this study, two nanobodies were selected and used to modulate the function of mouse P2X7. Interestingly, one of them potently blocks P2X7 while the other potentiates its activities. These nanobodies, termed 13A7 and 14D5, respectively, were used *in vivo* to validate the function of P2X7 in disease models. Systemic administration of 13A7 reduced inflammation in mouse models of allergic dermatitis and of glomerulonephritis. Conversely, 14D5 administrated *in vivo* aggravated disease scores in both animal models (47). Repeated administration of these selected anti-P2X7 nanobodies *in vivo* undoubtedly represents a novel means to study P2X7 functions in different pathophysiological situations.

## AAV-Nanobodies (AAVnano) Methodology

**FIGURE 1:**
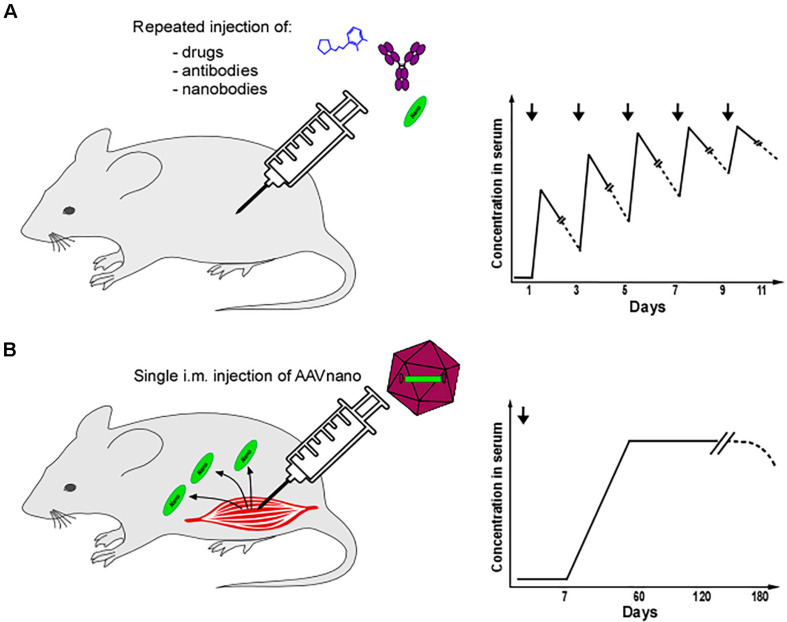
Comparison between repeated injection of drugs or antibodies/nanobodies and AAVnano methodology. Theoretical kinetics of the pharmacological agents in serum using different methodologies. **(A)** Small molecules (blue), antibodies (purple), or nanobodies (green) are generally injected using the i.p. or i.v. routes and necessitate repeated injections usually performed every 1–2 days. **(B)** Using the AAVnano methodology, a single injection of an AAV vector coding for the nanobody of interest is performed at day 0 using the i.m. route. The nanobody is then directly produced *in vivo* by the transduced muscle fibers. The nanobody is detectable in the serum 7–14 days post AAV injection and its concentration slowly increases until reaching a plateau 30–60 days post AAV injection. The nanobody produced *in vivo* can still be present in the serum 120 days post AAV injection.

We present and illustrate here the development of a novel methodology using adeno-associated viral vectors (AAV) encoding anti-P2X7 nanobodies for studying P2X7 function *in vivo*, notably in chronic situations. AAV vectors have widely been used for gene transfer *in vivo* to elicit long-term expression of the transgenic protein of interest. For instance, a single intramuscular injection of AAV encoding HIV-neutralizing antibodies resulted in their long-lasting systemic production in mice (48) and non-human primates (49). We implemented a similar AAV-mediated gene transfer method to produce *in situ* anti-P2X7 nanobodies with the aim to durably modulate P2X7 function *in vivo*. For that, we generated AAV vectors encoding a bivalent P2X7-antagonistic heavy chain antibody designated 13A7-hcAb (nanobody 13A7 fused to the hinge and Fc-domains of mouse IgG1) or a bivalent, half-life extended P2X7-potentiating nanobody dimer designated 14D5-dimHLE (dimer of nanobody 14D5 fused to the albumin-specific nanobody Alb8 conferring half-life extension) (47). The methodology that we developed, termed AAVnano, requires only a single intramuscular (i.m.) injection of the corresponding AAV vector to elicit long-term systemic expression of these nanobody constructs *in vivo* for at least 120 days (unpublished observations). This avoids the daily injection of nanobodies necessary to maximize the modulation of P2X7 functions and offers the possibility to inhibit or to potentiate P2X7 in chronic models such as chronic inflammation, autoimmune diseases, carcinogenesis, or tumor growth (Figure 1).

## Illustration of the AAVnano Methodology in a Tumor Model

**FIGURE 2:**
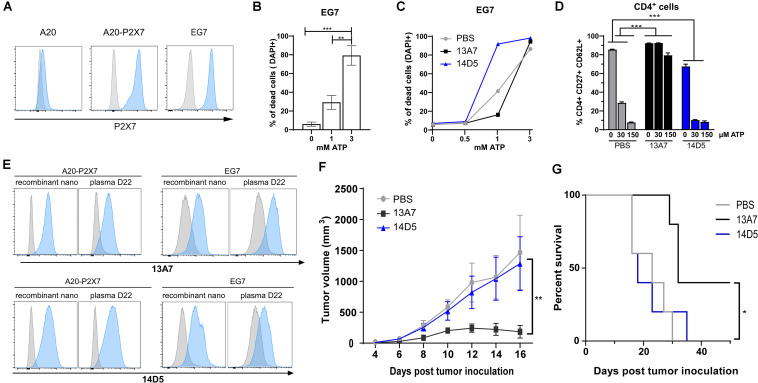
Using the AAVnano methodology to study the role of P2X7 in the EG7 tumor model. **(A)** C57BL/6 mouse EG7 T lymphoma cells expresses P2X7 at the cell membrane. EG7 T lymphoma cells, A20 B lymphoma cells and A20 cells stably transfected with P2X7 were incubated with fluorochrome conjugated P2X7-specific mAb Hano43 (blue) or an isotype control (gray). Bound antibodies were detected by flow cytometry. Similar results were obtained using the P2X7 specific polyclonal antibodies K1G (58). **(B)** EG7 cells are sensitive to ATP-induced cell death *in vitro*. EG7 cells were incubated overnight in culture medium with the indicated concentrations of ATP. Cell death was assessed by flow cytometry after staining with DAPI. The statistical analysis were performed using one-way ANOVA (*n* = 3, **p < 0.01; ***p < 0.001). **(C)** P2X7-specific nanobodies modulate ATP-induced death of EG7 cells. EG7 cells were preincubated for 1 h in the absence (PBS, gray) or presence of 1 μg/ml 13A7-hcAb (13A7, black) or 14D5-dimHLE (14D5, blue). Cells were then incubated overnight with 0, 0.5, 1, or 3 mM of ATP in culture medium and assayed for cell death using flow cytometry as in **(B)**. **(D)** Nanobodies produced *in situ* modulate P2X7-dependent shedding of CD62L and CD27 by CD4^+^ T cells. 8 weeks old C57BL/6 mice were injected i.m. with PBS (gray) or with AAV1 vector encoding either P2X7-antagonistic 13A7-hcAb (black) or P2X7-potentiating 14D5-dimHLE (blue). 3 weeks later (day 22), blood samples were collected and incubated for 20 min in PBS with the indicated concentration of ATP (0, 30, or 150 μM ATP). Cells were then stained with fluorochrome-conjugated antibodies against CD4, CD27, CD62L, before fixation and erythrocyte lysis. The percentages of CD4^+^ cells that co-express CD27 and CD62L were then evaluated by flow cytometry. The statistical analysis were performed using two-way ANOVA (*n* = 5, ***p < 0.001). Similar results were obtained in 3 independent experiments. **(E)** Plasma from AAV-injected mice contain circulating P2X7-specific nanobodies. A20-P2X7 and EG7 cells were incubated either with recombinant nanobodies (1 μg/ml) or with 10 μl of pooled plasma obtained at day 22 after AAV injection. Bound nanobodies were detected by flurochrome-conjugated secondary antibodies using flow cytometry. Histograms in gray correspond to the background staining obtained when using the sera from PBS injected animals. **(F,G)** P2X7 antagonist but not P2X7-agonist constructs inhibit EG7 lymphoma tumor growth *in vivo*. C57BL/6 mice were injected i.m. with 10^11^ AAV1 vectors encoding the P2X7-antagonistic 13A7-hcAb (13A7, black line), or the P2X7-potentiating 14D5-dimHLE (14D5, blue line). 23 days later, after having confirmed the production and the functionality of these constructs produced *in vivo* (see above **D,E**), 10^6^ EG7 lymphoma cells were inoculated subcutaneously in the left flanks. Tumors were monitored every other day during 50 days with a digital caliper, and the tumor volumes were estimated using the formula length × width × [(length + width)/2]. **(F)** Tumor volumes in each experimental group are shown for the first 16 days. The statistical analysis were performed using two-way ANOVA (*n* = 5, **p < 0.01). **(G)** Mouse survival is illustrated during the entire observation period. The statistical analysis was performed using Log-Rank tests (*n* = 5, *p < 0.05). Similar results were obtained in 3 independent experiments.

As discussed earlier, P2X7 plays complex and partially opposing roles in tumor growth and anti-tumor immune responses. To evaluate the potential utility of the AAVnano methodology, we chose to study the *in vivo* growth of the C57BL/6 mouse lymphoma cell line EG7 that naturally expresses significant surface levels of P2X7 (Figure 2A). We found that EG7 naturally expresses P2X7 at a level that is similar to the level detected at the surface of A20-P2X7 cells obtained after stable transfection of the BALB/c mouse B cell lymphoma A20 with an expression plasmid encoding full-length mouse P2X7 (50). To further characterize the functionality of P2X7 on EG7 cells, we verified their sensitivity to cell death induced by high concentrations of ATP (Figure 2B). As expected, addition of the recombinant nanobodies 13A7-hcAb or 14D5-dimHLE, respectively, inhibited and potentiated ATP induced-cell death, confirming the involvement and the functionality of P2X7 (Figure 2C).

Next, we aimed to study the situation *in vivo* in a context where P2X7 would be permanently and durably blocked or potentiated by 13A7-hcAb or 14D5-dimHLE nanobodies produced *in situ*. For that, AAV1 vectors encoding these constructs were injected intramuscularly (i.m.) into syngeneic naive C57BL/6 mice 3 weeks before inoculation of tumor cells. Before tumor inoculation, we obtained blood samples from the AAV injected mice. In order to evaluate the functional effects of nanobodies bound to circulating T cells *in vivo*, we incubated blood leukocytes obtained from these mice with ATP *ex vivo* and analyzed their sensitivity to ATP-induced shedding of CD27 and CD62L, a sensitive P2X7-dependent effect (15). As expected, CD4^+^ T lymphocytes from mice injected with AAV1 encoding P2X7-antagonistic 13A7-hcAb were resistant to ATP-induced CD27 and CD62L shedding while cells from the mice injected with AAV1 encoding the P2X7-potentiating 14D5-dimHLE showed enhanced sensitivity to ATP-induced shedding of CD27 and CD62L (Figure 2D). Further, indirect flow cytometry analyses *ex vivo* of EG7 and A20-P2X7 cells with plasma from these animals confirmed the presence of circulating nanobodies 3 weeks after AAV injection (Figure 2E).

Next, the EG7 tumor cells were injected subcutaneously, and tumor growth was monitored for 50 days. The results show that constant production of the P2X7 antagonistic 13A7-hcAb significantly limited EG7 tumor growth *in vivo* (Figure 2F). This treatment induced a complete tumor regression in 2 out of 5 mice and significantly improved the survival of treated mice (Figure 2G). These data are in line with previous studies showing that expression of P2X7 by tumor cells represents a trophic and pro-survival factor and that P2X7-blockade can inhibit tumor growth *in vivo* (24, 25, 28, 29). In contrast, potentiation of P2X7 function using AAV-mediated *in situ* expression of 14D5-dimHLE did not significantly influence tumor growth in this model (Figures 2F,G). One might have expected the opposite effect than 13A7-hcAb, e.g., promotion of tumor growth. On the other hand, P2X7 potentiation could also promote anti-tumor immune responses by mediating death of regulatory T cells (Tregs) by enhancing their sensitivity to NAD-mediated cell death (NICD). We indeed previously demonstrated that P2X7-dependent NICD of Tregs promotes an anti-tumor immune response in the same tumor model (15). The lack of an effect of 14D5-DimHLE on tumor growth could thus reflect balanced anti-tumoral/pro-tumoral effects in the tumor microenvironment. It will be interesting to determine whether potentiation of P2X7 can enhance anti-tumor immune response when combined with other treatments, e.g., anti-immune checkpoints therapy, immunogenic chemotherapies that increase the release of ATP, or antagonism of CD39/CD73-catalyzed hydrolysis of ATP to immunosuppressive adenosine (51, 52).

Overall, our data support the notion that the AAVnano methodology represents an additional strategy to study the function of P2X7 *in vivo*, allowing target validation in pathophysiological situations where P2X7 has been implicated, including cancer.

## Discussion and Perspectives Offered by the AAVnano Methodology to Study the Importance of P2X7 in Different Pathophysiological Situations

The AAVnano methodology presents several theoretical and practical advantages over other methodologies. As compared to chemical compounds, the nanobodies offer better target specificity. Indeed, nanobodies and antibodies in general are known for their excellent ability to discriminate between closely related targets belonging to the same protein family. This may prevent unwanted off-target effects. In the present study, the selected nanobodies bind specifically to mouse P2X7 and do not recognize the closest paralogs P2X4 and P2X1 (47). However, high specificity and lack of cross-reactivity with the P2X7 ortholog of other species may limit the use of a nanobody across species in translational studies. A similar limitation has also been observed with chemical compounds that sometimes display different modulating effects and pharmacological proprieties in humans and other animals.

Adeno-associated viral vectors are widely used in gene therapy settings and represent an efficient and safe approach to transfer a gene of interest into muscle cells and to elicit long-term systemic *in situ* production of a transgenic protein (48, 53). In our experiences, a single intramuscular administration of AAV (10^11^ viral genomes per mouse) coding for our engineered nanobody constructs was sufficient to elicit effective systemic levels of the blocking or the potentiating P2X7-specific nanobodies. The AAVnano methodology thus avoids daily administration of the corresponding recombinant nanobodies. Arguably, this may additionally provide more favorable pharmacokinetics by maintaining a stable saturating concentration of the desired nanobody *in vivo* and by avoiding peak/decline cycles obtained after repeated injection of a recombinant antibody (Figure 1). This may favor a better bioavailability and tissue penetration, which needs to be confirmed experimentally in future studies. AAVnano may provide a nanobody delivery methodology similar to the one obtained with osmotic pump drug delivery systems, which allow a stable equilibrium of the pharmacological agent *in vivo* at a plateau phase (Figure 1). However, as systemic production of transgenic protein *in situ* using AAV vectors is known to follow slow kinetics, with the apparition of the protein of interest 1 to 2 weeks post-transduction, the equilibrium phase is probably not obtained *in vivo* before 2 to 3 weeks (54–56). Hence, the AAVnano methodology is intended to be used in prophylactic models, as in the present study, rather than in a therapeutic setting. However, the kinetics of transgenic protein production can be considerably accelerated using recently developed self-complementary AAV (scAAV) vectors that would be more compatible with therapeutic/interventional protocols (56).

One of the features associated with the AAVnano methodology is the permanent and long-term modulation of P2X7 *in vivo* without any possibility to halt the treatment. While this is adapted to the evaluation of P2X7 functions in chronic diseases, this may appear as an inconvenient in other situations. Additionally, long-term P2X7 modulation (i.e., activation or potentiation) may have unexpected effects on the general physiology of the mice by causing, for instance, immune dysregulation, alteration of barrier functions, or alteration of the microbiota. Knockout mice may possibly not be affected to the same extent by P2X7 deficiency as compensatory mechanisms are presumably induced during embryogenesis, as often observed in knockout animals. In this regard, AAVnano methodology may reveal so far unexpected physiological functions of P2X7 that may be interesting to further explore. One way to avoid such long-term effects would be to use inducible promoters, instead of the ubiquitous CBA promoter used in this study. Such an inducible system, combined with AAV-mediated gene transfer, would allow controlled and timely delivery of the selected nanobody to modulate P2X7 only when required. This type of inducible delivery would be compatible with both, a prophylactic scheme as well as therapeutic/interventional protocols.

Beyond the cancer field, the AAVnano methodology may be further exploited in target-validation studies and in other disease models where P2X7 has been incriminated. Examples are inflammatory models such as acute and chronic experimental colitis, or autoimmune diseases like rheumatoid arthritis, lupus, or experimental allergic encephalitis. Interestingly, besides skeletal muscles, AAV vectors can be used to transduce efficiently a variety of other tissues including heart, liver, kidney, retina, and the central nervous system (CNS). For example, it is conceivable to use stereotaxic injection of AAV9 vectors into the brain to induce expression of the modulating P2X7-specific nanobodies in the CNS. Since P2X7 is expressed in the CNS, particularly by microglial cells and astrocytes, AAVnano may help to elucidate the role of P2X7 in the pathophysiology of neurological disorders like epilepsy, Alzheimer’s disease, multiple sclerosis, amyotrophic lateral sclerosis, age-related macular degeneration, or cerebral artery occlusion (57). Apart from using tissue-specific conditional P2X7 knockout models, the role of P2X7 in these diseases remains difficult to evaluate as the use of antibodies is limited by the blood-brain barrier. AAVnano may offer a possibility to circumvent this difficulty by exploiting the efficiency of AAV vectors to transduce different regions of the CNS, combined with the good tissue penetration and bioavailability offered by nanobodies. Hence, the AAVnano methodology may pave the way to a better understanding of the role of P2X7 not only in the tumor context, but also in chronic inflammatory/autoimmune diseases and neurological disorders. This may further help the community to evaluate the importance of P2X7 in pathophysiology and as a therapeutic target.

## Data Availability Statement

The datasets generated for this study are available on request to the corresponding author.

## Ethics Statement

The animal study was reviewed and approved by Comité d’Ethique NOrmandie en Matière d’EXpérimentation Animale (CENOMEXA).

## Author Contributions

MD, FH, FK-N, and SA conceptualization. MD, AS, CA, and SA methodology. MD, CA, and SA investigation. MD, FK-N, and SA writing – original draft. MD, AS, CA, HG, RH, CP-E, AE, WS, FH, FK-N, and SA writing – review and editing. FK-N and SA funding acquisition. FH, FK-N, and SA Resources. CA, FK-N, and SA supervision. All authors contributed to the article and approved the submitted version.

## Conflict of Interest

The authors declare that the research was conducted in the absence of any commercial or financial relationships that could be construed as a potential conflict of interest.

## References

[1] Burnstock G. Purinergic signalling. *Br J Pharmacol.* (2009) 147:S172–81. 10.1038/sj.bjp.0706429 16402102 PMC1760723

[2] Di Virgilio F Schmalzing G Markwardt F. The elusive P2X7 macropore. *Trends Cell Biol.* (2018) 28:392–404. 10.1016/j.tcb.2018.01.005 29439897

[3] Di Virgilio F Giuliani AL Vultaggio-Poma V Falzoni S Sarti AC. Non-nucleotide agonists triggering P2X7 receptor activation and pore formation. *Front Pharmacol.* (2018) 9:39. 10.3389/fphar.2018.00039 29449813 PMC5799242

[4] Peverini L Beudez J Dunning K Chataigneau T Grutter T. New insights into permeation of large cations through ATP-gated P2X receptors. *Front Mol Neurosci.* (2018) 11:265. 10.3389/fnmol.2018.00265 30108481 PMC6080412

[5] Adriouch S Haag F Boyer O Seman M Koch-Nolte F. Extracellular NAD(+): a danger signal hindering regulatory T cells. *Microbes Infect.* (2012) 14:1284–92. 10.1016/j.micinf.2012.05.011 22634347

[6] Rissiek B Haag F Boyer O Koch-Nolte F Adriouch S. ADP-ribosylation of P2X7: a matter of life and death for regulatory T cells and natural killer T cells. *Curr Top Microbiol Immunol.* (2015) 384:107–26. 10.1007/82_2014_420 25048544

[7] Gilbert S Oliphant C Hassan S Peille A Bronsert P Falzoni S ATP in the tumour microenvironment drives expression of nfP2X 7, a key mediator of cancer cell survival. *Oncogene.* (2019) 38:194–208. 10.1038/s41388-018-0426-6 30087439 PMC6328436

[8] Pellegatti P Raffaghello L Bianchi G Piccardi F Pistoia V Di Virgilio F. Increased level of extracellular ATP at tumor sites: In vivo imaging with plasma membrane luciferase. *PLoS One.* (2008) 3:e2599. 10.1371/journal.pone.0002599 18612415 PMC2440522

[9] Young CNJ Górecki DC. P2RX7 purinoceptor as a therapeutic target-the second coming? *Front Chem.* (2018) 6:248. 10.3389/fchem.2018.00248 30003075 PMC6032550

[10] Perregaux DG McNiff P Laliberte R Conklyn M Gabel CA. ATP acts as an agonist to promote stimulus-induced secretion of IL-1 beta and IL-18 in human blood. *J Immunol.* (2000) 165:4615–23. 10.4049/jimmunol.165.8.4615 11035104

[11] Rissiek B Haag F Boyer O Koch-Nolte F Adriouch S. P2X7 on mouse T cells: one channel, many functions. *Front Immunol.* (2015) 6:204. 10.3389/fimmu.2015.00204 26042119 PMC4436801

[12] Sommer A Kordowski F Büch J Maretzky T Evers A Andrä J Phosphatidylserine exposure is required for ADAM17 sheddase function. *Nat Commun.* (2016) 7:11523. 10.1038/ncomms11523 27161080 PMC4866515

[13] Stark R Wesselink TH Behr FM Kragten NAM Arens R Koch-Nolte F TRM maintenance is regulated by tissue damage via P2RX7. *Sci Immunol.* (2018) 3:eaau1022. 10.1126/SCIIMMUNOL.AAU1022 30552101

[14] Rissiek B Lukowiak M Raczkowski F Magnus T Mittrücker HW Koch-Nolte F. Blockade of murine ARTC2.2 during cell preparation preserves the vitality and function of liver tissue-resident memory T cells. *Front Immunol.* (2018) 9:1580. 10.3389/fimmu.2018.01580 30038627 PMC6046629

[15] Hubert S Rissiek B Klages K Huehn J Sparwasser T Haag F Extracellular NAD+ shapes the Foxp3+ regulatory T cell compartment through the ART2-P2X7 pathway. *J Exp Med.* (2010) 207:2561–8. 10.1084/jem.20091154 20975043 PMC2989765

[16] Di Virgilio F Dal Ben D Sarti AC Giuliani AL Falzoni S. The P2X7 receptor in infection and inflammation. *Immunity.* (2017) 47:15–31. 10.1016/j.immuni.2017.06.020 28723547

[17] Adinolfi E Giuliani AL De Marchi E Pegoraro A Orioli E Di Virgilio F. The P2X7 receptor: a main player in inflammation. *Biochem Pharmacol.* (2018) 151:234–44. 10.1016/j.bcp.2017.12.021 29288626

[18] Hofman P Cherfils-Vicini J Bazin M Ilie M Juhel T Hébuterne X Genetic and pharmacological inactivation of the purinergic P2RX7 receptor dampens inflammation but increases tumor incidence in a mouse model of colitis-associated cancer. *Cancer Res.* (2015) 75:835–45. 10.1158/0008-5472.CAN-14-1778 25564520

[19] Labasi JM Petrushova N Donovan C McCurdy S Lira P Payette MM Absence of the P2X 7 receptor alters leukocyte function and attenuates an inflammatory response. *J Immunol.* (2002) 168:6436–45. 10.4049/jimmunol.168.12.6436 12055263

[20] Sharp AJ Polak PE Simonini V Lin SX Richardson JC Bongarzone ER P2x7 deficiency suppresses development of experimental autoimmune encephalomyelitis. *J Neuroinflammation.* (2008) 5:33. 10.1186/1742-2094-5-33 18691411 PMC2518548

[21] Park J-H Kim Y-C. P2X7 receptor antagonists: a patent review. (2010–2015). *Expert Opin Ther Pat.* (2017) 27:257–67. 10.1080/13543776.2017.1246538 27724045

[22] Ghiringhelli F Apetoh L Tesniere A Aymeric L Ma Y Ortiz C Activation of the NLRP3 inflammasome in dendritic cells induces IL-1β–dependent adaptive immunity against tumors. *Nat Med.* (2009) 15:1170–8. 10.1038/nm.2028 19767732

[23] Adinolfi E Capece M Franceschini A Falzoni S Giuliani AL Rotondo A Accelerated tumor progression in mice lacking the ATP receptor P2X7. *Cancer Res.* (2015) 75:635–44. 10.1158/0008-5472.CAN-14-1259 25542861

[24] Adinolfi E Callegari MG Ferrari D Bolognesi C Minelli M Wieckowski MR Basal activation of the P2X7 ATP receptor elevates mitochondrial calcium and potential, increases cellular ATP levels, and promotes serum-independent growth. *Mol Biol Cell.* (2005) 16:3260–72. 10.1091/mbc.e04-11-1025 15901833 PMC1165409

[25] Adinolfi E Callegari MG Cirillo M Pinton P Giorgi C Cavagna D Expression of the P2X7 receptor increases the Ca2+ content of the endoplasmic reticulum, activates NFATc1, and protects from apoptosis. *J Biol Chem.* (2009) 284:10120–8. 10.1074/jbc.M805805200 19204004 PMC2665066

[26] Roger S Jelassi B Couillin I Pelegrin P Besson P Jiang L-H. Understanding the roles of the P2X7 receptor in solid tumour progression and therapeutic perspectives. *Biochim Biophys Acta.* (2015) 1848:2584–602. 10.1016/j.bbamem.2014.10.029 25450340

[27] Roger S Pelegrin P. P2X7 receptor antagonism in the treatment of cancers. *Expert Opin Investig Drugs.* (2011) 20:875–80. 10.1517/13543784.2011.583918 21619470

[28] Adinolfi E Raffaghello L Giuliani AL Cavazzini L Capece M Chiozzi P Expression of P2X7 receptor increases in vivo tumor growth. *Cancer Res.* (2012) 72:2957–69. 10.1158/0008-5472.CAN-11-1947 22505653

[29] Hattori F Ohshima Y Seki S Tsukimoto M Sato M Takenouchi T Feasibility study of B16 melanoma therapy using oxidized ATP to target purinergic receptor P2X7. *Eur J Pharmacol.* (2012) 695:20–6. 10.1016/j.ejphar.2012.09.001 22981895

[30] Solle M Labasi J Perregaux DG Stam E Petrushova N Koller BH Altered cytokine production in mice lacking P2X(7) receptors. *J Biol Chem.* (2001) 276:125–32. 10.1074/jbc.M006781200 11016935

[31] Chessell IP Hatcher JP Bountra C Michel AD Hughes JP Green P Disruption of the P2X7 purinoceptor gene abolishes chronic inflammatory and neuropathic pain. *Pain.* (2005) 114:386–96. 10.1016/j.pain.2005.01.002 15777864

[32] Nicke A Kuan YH Masin M Rettinger J Marquez-Klaka B Bender O A functional P2X7 splice variant with an alternative transmembrane domain 1 escapes gene inactivation in P2X7 knock-out mice. *J Biol Chem.* (2009) 284:25813–22. 10.1074/jbc.M109.033134 19546214 PMC2757983

[33] Taylor SR Gonzalez-Begne M Sojka DK Richardson JC Sheardown SA Harrison SM Lymphocytes from P2X7-deficient mice exhibit enhanced P2X7 responses. *J Leukoc Biol.* (2009) 85:978–86. 10.1189/jlb.0408251 19276178 PMC2698584

[34] Masin M Young C Lim K Barnes SJ Xu XJ Marschall V Expression, assembly and function of novel C-terminal truncated variants of the mouse P2X7 receptor: re-evaluation of P2X7 knockouts. *Br J Pharmacol.* (2012) 165:978–93. 10.1111/j.1476-5381.2011.01624.x 21838754 PMC3312493

[35] Chen L Brosnan CF. Exacerbation of experimental autoimmune encephalomyelitis in P2X7R-/- mice: evidence for loss of apoptotic activity in lymphocytes. *J Immunol.* (2006) 176:3115–26. 10.4049/jimmunol.176.5.3115 16493071

[36] Bartlett R Stokes L Sluyter R. The P2X7 receptor channel: recent developments and the use of P2X7 antagonists in models of disease. *Pharmacol Rev.* (2014) 66:638–75. 10.1124/pr.113.008003 24928329

[37] Kaczmarek-Hajek K Zhang J Kopp R Grosche A Rissiek B Saul A Re-evaluation of neuronal P2X7 expression using novel mouse models and a P2X7-specific nanobody. *Elife.* (2018) 7:e36217. 10.7554/eLife.36217 30074479 PMC6140716

[38] Faliti CE Gualtierotti R Rottoli E Gerosa M Perruzza L Romagnani A P2X7 receptor restrains pathogenic Tfh cell generation in systemic lupus erythematosus. *J Exp Med.* (2019) 216:317–36. 10.1084/jem.20171976 30655308 PMC6363434

[39] Arulkumaran N Unwin RJ Tam FW. A potential therapeutic role for P2X7 receptor (P2X7R) antagonists in the treatment of inflammatory diseases. *Expert Opin Investig Drugs.* (2011) 20:897–915. 10.1517/13543784.2011.578068 21510825 PMC3114873

[40] Matute C Torre I Pérez-Cerdá F Pérez-Samartín A Alberdi E Etxebarria E P2X(7) receptor blockade prevents ATP excitotoxicity in oligodendrocytes and ameliorates experimental autoimmune encephalomyelitis. *J Neurosci.* (2007) 27:9525–33. 10.1523/JNEUROSCI.0579-07.2007 17728465 PMC6673129

[41] Dell’Antonio G Quattrini A Dal Cin E Fulgenzi A Ferrero ME. Antinociceptive effect of a new P(2Z)/P2X7 antagonist, oxidized ATP, in arthritic rats. *Neurosci Lett.* (2002) 327:87–90. 10.1016/s0304-3940(02)00385-3 12098642

[42] Taylor SR Turner CM Elliott JI McDaid J Hewitt R Smith J P2X7 deficiency attenuates renal injury in experimental glomerulonephritis. *J Am Soc Nephrol.* (2009) 20:1275–81. 10.1681/ASN.2008060559 19389853 PMC2689903

[43] Li Z Krippendorff BF Sharma S Walz AC Lavé T Shah DK. Influence of molecular size on tissue distribution of antibody fragments. *MAbs.* (2016) 8:113–9. 10.1080/19420862.2015.1111497 26496429 PMC5040103

[44] Wesolowski J Alzogaray V Reyelt J Unger M Juarez K Urrutia M Single domain antibodies: promising experimental and therapeutic tools in infection and immunity. *Med Microbiol Immunol.* (2009) 198:157–74. 10.1007/s00430-009-0116-7 19529959 PMC2714450

[45] Muyldermans S. Nanobodies: natural single-domain antibodies. *Annu Rev Biochem.* (2013) 82:775–97. 10.1146/annurev-biochem-063011-092449 23495938

[46] Bannas P Hambach J Koch-Nolte F. Nanobodies and nanobody-based human heavy chain antibodies as antitumor therapeutics. *Front Immunol.* (2017) 8:1603. 10.3389/fimmu.2017.01603 29213270 PMC5702627

[47] Danquah W Catherine MS Rissiek B Pinto C Arnau SP Amadi M Nanobodies that block gating of the P2X7 ion channel ameliorate inflammation. *Sci Transl Med.* (2016) 8:366ra162. 10.1126/scitranslmed.aaf8463 27881823

[48] Balazs AB Ouyang Y Hong CM Chen J Nguyen SM Rao DS Vectored immunoprophylaxis protects humanized mice from mucosal HIV transmission. *Nat Med.* (2014) 20:296–300. 10.1038/nm.3471 24509526 PMC3990417

[49] Saunders KO Wang L Gordon Joyce M Yang Z-Y Balazs AB Cheng C Broadly neutralizing human immunodeficiency virus type 1 antibody gene transfer protects non-human primates from mucosal simian-human immunodeficiency virus infection. *J Virol.* (2015) 89:8334–45. 10.1128/JVI.00908-15 26041300 PMC4524228

[50] Adriouch S Bannas P Schwarz N Fliegert R Guse AH Seman M ADP−ribosylation at R125 gates the P2X7 ion channel by presenting a covalent ligand to its nucleotide binding site. *FASEB J.* (2008) 22:861–9. 10.1096/fj.07-9294com 17928361

[51] Garg AD Krysko DV Vandenabeele P Agostinis P. Extracellular ATP and P2X7 receptor exert contextspecific immunogenic effects after immunogenic cancer cell death. *Cell Death Dis.* (2016) 7:e2097. 10.1038/cddis.2015.411 26890136 PMC5399185

[52] Hammami A Allard D Allard B Stagg J. Targeting the adenosine pathway for cancer immunotherapy. *Semin Immunol.* (2019) 42:101304. 10.1016/j.smim.2019.101304 31604539

[53] Saunders KO Wang L Joyce MG Yang ZY Balazs AB Cheng C Broadly neutralizing human immunodeficiency virus type 1 antibody gene transfer protects non-human primates from mucosal simian-human immunodeficiency virus infection. *J Virol.* (2015) 89:8334–45. 26041300 10.1128/JVI.00908-15 PMC4524228

[54] Adriouch S Franck E Drouot L Bonneau C Jolinon N Salvetti A Improved immunological tolerance following combination therapy with CTLA-4/Ig and AAV-mediated PD-L1/2 muscle gene transfer. *Front Microbiol.* (2011) 2:199. 10.3389/fmicb.2011.00199 22046170 PMC3202221

[55] Hardet R Chevalier B Dupaty L Naïmi Y Riou G Drouot L Oral-tolerization prevents immune responses and improves transgene persistence following gene transfer mediated by adeno-associated viral vector. *Mol Ther.* (2016) 24:87–95. 10.1038/mt.2015.146 26265250 PMC4754539

[56] McCarty DM. Self-complementary AAV vectors; advances and applications. *Mol Ther.* (2008) 16:1648–56. 10.1038/mt.2008.171 18682697

[57] Kanellopoulos JM Delarasse C. Pleiotropic roles of P2X7 in the central nervous system. *Front Cell Neurosci.* (2019) 13:401. 10.3389/fncel.2019.00401 31551714 PMC6738027

[58] Seman M Adriouch S Scheuplein F Krebs C Freese D Glowacki G NAD-induced T cell death: ADP-ribosylation of cell surface proteins by ART2 activates the cytolytic P2X7 purinoceptor. *Immunity.* (2003) 19:571–82. 10.1016/S1074-7613(03)00266-8 14563321

